# Structure of the human RNA polymerase I elongation complex

**DOI:** 10.1038/s41421-021-00335-5

**Published:** 2021-10-20

**Authors:** Dan Zhao, Weida Liu, Ke Chen, Zihan Wu, Huirong Yang, Yanhui Xu

**Affiliations:** 1 Fudan University Shanghai Cancer Center, Institutes of Biomedical Sciences, State Key Laboratory of Genetic Engineering, Shanghai Key Laboratory of Radiation Oncology, and Shanghai Key Laboratory of Medical Epigenetics, Shanghai Medical College of Fudan University, Shanghai, China; 2 The International Co-laboratory of Medical Epigenetics and Metabolism, Ministry of Science and Technology of China, Department of Systems Biology for Medicine, School of Basic Medical Sciences, Shanghai Medical College of Fudan University, Shanghai, China; 3 Human Phenome Institute, Collaborative Innovation Center of Genetics and Development, School of Life Sciences, Fudan University, Shanghai, China; 4 State Key Laboratory of Reproductive Regulation and Breeding of Grassland Livestock, School of Life Sciences, Inner Mongolia University, Hohhot, Inner Mongolia China

**Keywords:** Cryoelectron microscopy, Transcription

## Abstract

Eukaryotic RNA polymerase I (Pol I) transcribes ribosomal DNA and generates RNA for ribosome synthesis. Pol I accounts for the majority of cellular transcription activity and dysregulation of Pol I transcription leads to cancers and ribosomopathies. Despite extensive structural studies of yeast Pol I, structure of human Pol I remains unsolved. Here we determined the structures of the human Pol I in the pre-translocation, post-translocation, and backtracked states at near-atomic resolution. The single-subunit peripheral stalk lacks contacts with the DNA-binding clamp and is more flexible than the two-subunit stalk in yeast Pol I. Compared to yeast Pol I, human Pol I possesses a more closed clamp, which makes more contacts with DNA. The Pol I structure in the post-cleavage backtracked state shows that the C-terminal zinc ribbon of RPA12 inserts into an open funnel and facilitates “dinucleotide cleavage” on mismatched DNA–RNA hybrid. Critical disease-associated mutations are mapped on Pol I regions that are involved in catalysis and complex organization. In summary, the structures provide new sights into human Pol I complex organization and efficient proofreading.

## Introduction

Among the three eukaryotic RNA polymerases (Pol I, Pol II, and Pol III)^1^, Pol I accounts for up to 60% of cellular transcriptional activity^2,3^. The human Pol I (hPol I) is located in the nucleolus and synthesizes 47S pre-ribosome RNA^4^. The 47S pre-ribosome RNA is further processed into mature 18S, 5.8S, and 28S ribosomal RNAs (rRNAs), which compose the RNA components of ribosomes together with 5S RNA synthesized by Pol III^5^. The Pol I-mediated pre-rRNA transcription is strictly required for ribosome biogenesis^2^. Dysregulation of Pol I transcription is associated with human diseases and the Pol I transcription machinery is considered to be the drug target for anticancer therapy^6–9^.

The hPol I consists of 13 subunits and the yeast Pol I (yPol I) has similar counterparts with an additional yeast-specific subunit, A14. Among the 10 core subunits of the hPol I, five subunits (RPABC1, RPABC2, RPABC3, RPABC4, and RPABC5) are shared by the three RNA polymerases (Pol I, II, and III) and two subunits (RPAC1 and RPAC2) are shared by Pol I and Pol III, indicating a highly conserved catalytic core^10^. The general transcription factors TFIIE and TFIIF are dissociable and serve as regulatory complexes in Pol II, while the TFIIF-/TFIIE-like subunits are evolved as bona fide constitutive subunits, PAF53/PAF49 in Pol I and C37/C53 in Pol III^11^. Additionally, the yPol I stalk is formed by A14/A43 heterodimer, whereas the functional hPol I stalk consists of only one subunit, RPA43, and the A14 counterpart has not been identified.

The structure of yPol I has been elaborately studied in the past decades. The overall architecture of yPol I was reported in early studies^12–14^. Recent studies determined yPol I structures in distinct nucleotide-binding states^15–18^. It remains incompletely understood how hPol I is assembled and what the differences are between hPol I and yPol I. Here we present cryo-electron microscopy (cryo-EM) structures of hPol I elongation complex (EC) in the pre-translocation, post-translocation, and backtracked states. These structures reveal hPol I-specific structural features and molecular mechanism of backtracking and RNA cleavage of the mismatched DNA–RNA hybrid for proofreading.

## Results

### Complex assembly and structure determination of the hPol I ECs

**Fig. 1:**
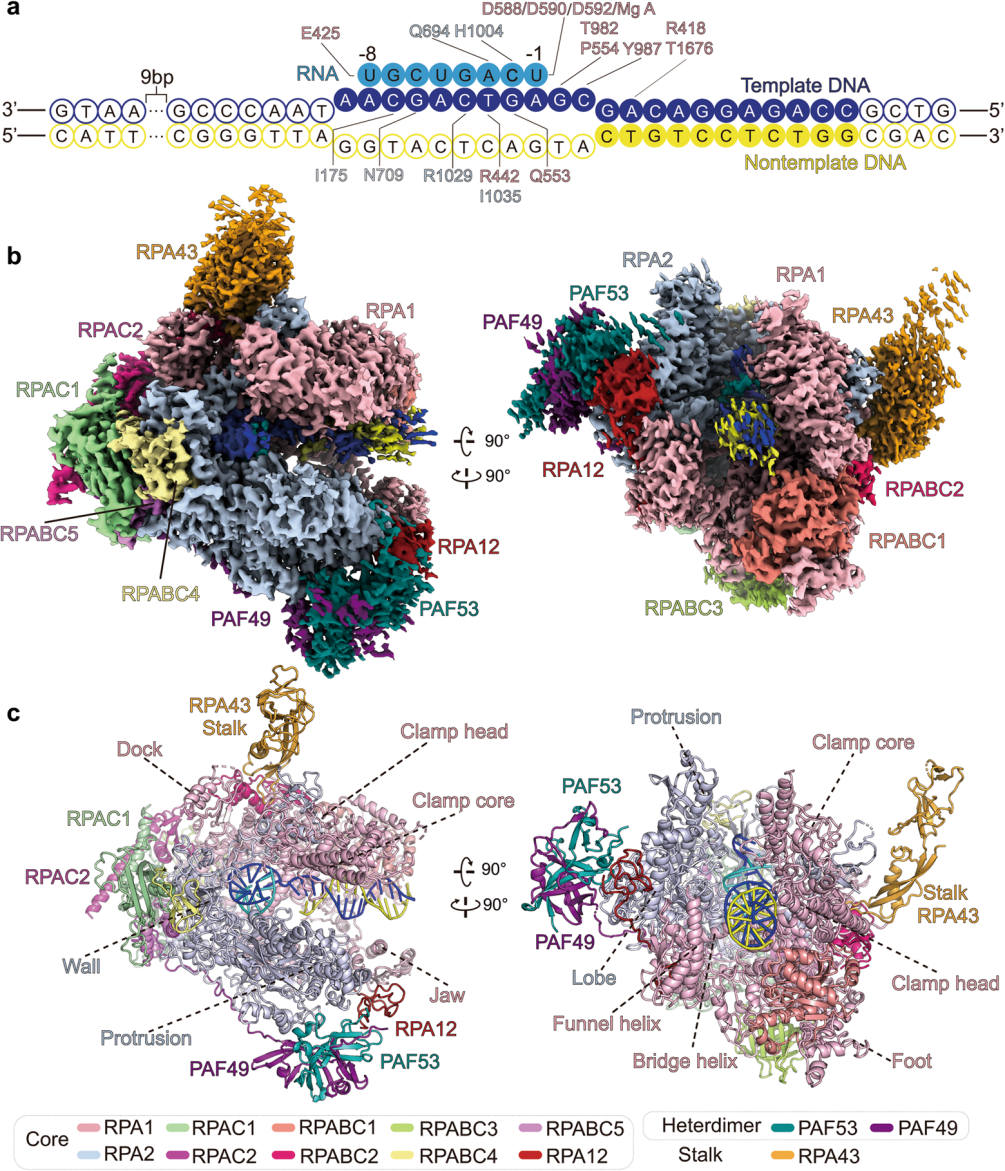
Cryo-EM structure of the hPol I EC^post^. **a** Schematic model of the DNA–RNA scaffold used in EC^post^. RNA is numbered relative to the NTP addition site in the elongation complex. The template DNA is shown in blue, the nontemplate DNA in yellow, and RNA in cyan. The bases that built in our model are shown with color-filled circles. Residues involved in contacting nucleotides are indicated. **b**, **c** Cryo-EM map (**b**) and structural model (**c**) of the hPol I EC^post^ in two different views. Color scheme is indicated and used in all the figures below.

The 13-subunit hPol I was overexpressed in Expi293F cells and purified to homogeneity for structural and biochemical analyses (Supplementary Fig. S1a). The purified complex exhibited DNA-dependent RNA elongation activity on DNA template and RNA cleavage activity on a mismatched DNA–RNA hybrid, indicative of a functional Pol I (Supplementary Fig. S1b). To elucidate the molecular mechanism of Pol I-mediated transcription elongation, we assembled three complexes mimicking ECs in the pre-translocation, post-translocation, and backtracked states, respectively (Fig. 1a and Supplementary Table S1). The Pol I EC in the post-translocation state (EC^post^) was assembled by incubating the purified Pol I and a DNA–RNA hybrid scaffold consisting of 11 mismatched DNA base pairs and 8-nt RNA^19^. The EC in the pre-translocation state (EC^pre^) was assembled by adding CMPCPP (cytidine-5′-[(α, β)-methyleno]-triphosphate), a non-hydrolysable nucleotide, to the EC^post^. The DNA–RNA scaffold used in the assembly of EC in the backtracked state (EC^bt^) consists of an additional mismatched DNA–RNA base pair at −1 site (relative to the NTP addition site) (Supplementary Table S2).

The structures of Pol I EC in the three states were determined using cryo-EM single-particle reconstruction (Supplementary Fig. S2 and Tables S1 and S3). The cryo-EM maps of EC^post^, EC^pre^, and EC^bt^ were refined to 2.8, 2.9, and 3.0 Å resolutions, respectively. The cryo-EM maps of the three structures reveal well-ordered core complex and support unambiguous tracing of residues and nucleotides (Fig. 1c and Supplementary Fig. S3). The structural models were manually built aided by the yPol I structures^16,18^ as template and secondary structure prediction. The peripheral regions were relatively flexible and the structural model was built by fitting structural templates into the cryo-EM maps, followed by manual adjustment.

### Overall structure of hPol I EC in the post-translocation state

The Pol I structures in the three states adopt almost identical overall fold except for distinct organization of nucleic acids within the catalytic center and slight differences in modular organization of EC^bt^ (Supplementary Fig. S4a). The structure of EC^post^ will be discussed below in analyzing the shared structure features (Fig. 1 and Supplementary Video S1).

The cryo-EM map of Pol I EC^post^ reveals a rigid globular core formed by ten core subunits (Fig. 1b). The two Pol I-specific large subunits, RPA1 and RPA2, create the central DNA-binding cleft and are surrounded by the rest of subunits. The five Pol I/II/III-shared components (RPABC1, RPABC2, RPABC3, RPABC4, and RPABC5) are surrounding subunits and integrated into the core module through binding RPA1 and RPA2. The RPAC1 and RPAC2, two shared subunits of Pol I/III, interact with each other and pack against the wall of Pol I. The TFIIS-like subunit RPA12 has an N-terminal zinc ribbon (N-ribbon), which packs against the dimerization domain of TFIIF-like PAF53/PAF49 heterodimer and the lobe of RPA2. The C-terminal ribbon (C-ribbon) of RPA12 inserts into the funnel in the backtracked state, consistent with its role in proofreading (detailed below).

**Fig. 2:**
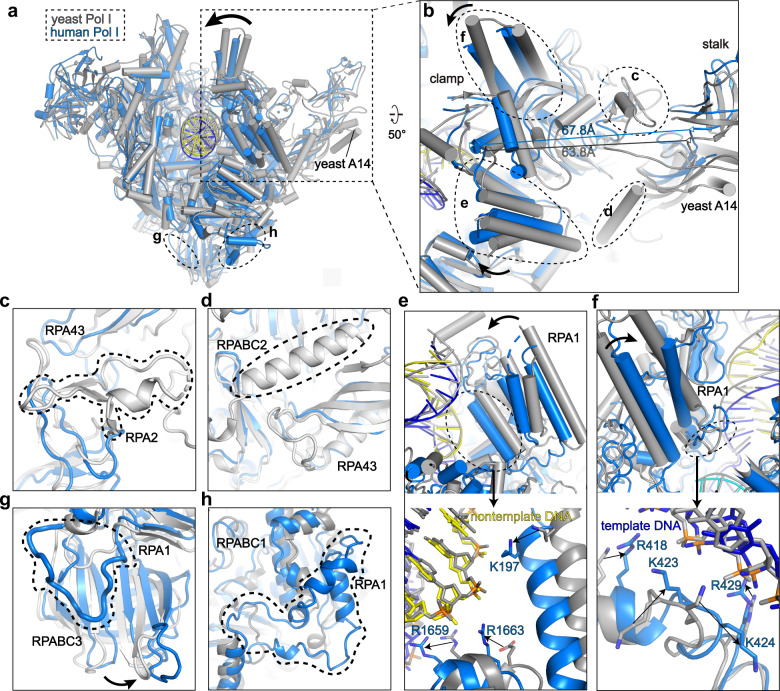
Structural differences between hPol I and yPol I. **a** Structural comparison of hPol I (marine) and yPol I (gray) (PDB: 5M3F)^16^ in the EC^post^ state. **b**–**f** Close-up views of structural differences around the clamp between hPol I and yPol I and the differences are indicated with arrows. The hPol I subunits are labeled. Two dashed circles in **c**, **d** indicate the residues 1133–1168 of A135 and residues 52–68 of Rpb6 in yPol I, respectively. These two regions do not exist in hPol I. Two different views of the structural difference in the clamp are shown in the top panels of **e** and **f** and more details are shown at the bottom panels. Two dashed circles in **g**, **h** indicate residues 721–732 and 1078–1131 insertions of RPA1 that exist in hPol I but not in yPol I, respectively.

The cryo-EM map reveals relatively weak density around the three peripheral subunits (Fig. 1b). We performed glutaraldehyde crosslinking to prepare EC^post^ followed by structure determination, generating improved cryo-EM density around RPA43, PAF53, and PAF49 (Supplementary Fig. S2). Structural models were built by docking the structural templates of these subunits in yPol I into the cryo-EM map with the aid of AlphaFold prediction^20^ followed by manual adjustment. The flexible stalk consists of one subunit (RPA43) and lacks the yeast counterpart A14 (Fig. 2a and Supplementary Fig. S5a). The N-terminal dimerization domains of PAF53 and PAF49 interact with each other and together form a conserved triple β barrel-like fold, similar to the dimerization domain of TFIIF^21^. The linker region of PAF49 winds over RPA2, RPAC1, and RPABC5 and facilitates positioning of the PAF53–PAF49 heterodimer on the lobe. The positively-charged C-terminal tail of PAF49, the TFIIE-like C-terminal tandem winged helix domain and the linker region of PAF53 were not observed due to their flexibility.

### The hPol I has a more closed DNA-binding clamp than yPol I

The hPol I EC adopts an overall architecture generally similar to that of yPol I EC^16^ (Fig. 2a and Supplementary Video S2), consistent with the high sequence similarity of the core subunits^13,14^. Structural comparison shows that the central core and DNA–RNA hybrid are well superimposed and the major difference exists in the conformational arrangement of the clamp (Fig. 2a, b). The yPol I has a relatively closer clamp–stalk association with the two modules stably bridged by yPol I-specific insertions, residues 1133–1168 of A135 (human RPA2 counterpart) and residues 52–68 of Rpb6 (human RPABC2 counterpart) (Fig. 2b–d and Supplementary Fig. S6b, c). The intermodular association is further supported by the N-terminal region (residues 12–24) of the yPol I-specific subunit A14 (Fig. 2a, b and Supplementary Fig. S5a). Due to the lack of stabilizations by equivalent regions, the stalk and clamp are more separated in hPol I, as measured by ~68 Å between residues P125 (RPA43 in hPol I) of stalk and R101 (RPA1 in hPol I) of clamp, compared to the separation of equivalent residues by ~64 Å in yPol I (Fig. 2b). As a result, the clamp in hPol I is positioned closer (by ~4 Å) to the DNA than that in yPol I, generating a more closed DNA entry cleft (Fig. 2b, e, f). Positively-charged residues K197, R1663, R1659 (Fig. 2e), R418, K423, K424, and R429 (Fig. 2f) of the clamp are brought into close contact with the phosphate groups of both template and nontemplate strands and may stabilize the EC during transcription elongation. In contrast, yPol I has much less clamp–DNA contacts. Other structural differences were observed in the two human-specific insertions (residues 721–732 and 1078–1131) in RPA1 and slight positional shifts of their adjacent domains (Fig. 2g, h and Supplementary Fig. S6a).

### The single-subunit stalk of hPol I

In yPol I, A43 and A14 subunits form the stalk (Supplementary Fig. S5a). It is known that A43 interacts with transcription factor Rrn3^22^, an essential transcription initiation factor conserved in human and yeast^23–26^. However, A14 counterpart has not been identified in hPol I. The purified hPol I complex was catalytically active in our in vitro transcription assay (Supplementary Fig. S1a), suggesting that A14 is dispensable for hPol I elongation activity.

The cryo-EM map of the stalk is relatively weak, consistent with the relatively small contact surface between RPA43 and the Pol I core (Fig. 1b). Similar to Rpb7 of Pol II^27^ and C25 of Pol III^28^, the human stalk subunit RPA43 adopts an extended fold and consists of a Tip domain and an oligonucleotide binding-fold domain (Fig. 1c)^14^. The predicted C-terminal positively-charged tail of RPA43 is invisible. The yeast A43–A14 heterodimer has been believed to direct the dimerization of Pol I^14^. However, no obvious dimerization of hPol I was observed (Supplementary Fig. S1c).

The above structural differences between hPol I and yPol I may reflect distinct functional requirements of Pol I in the two species. For example, the clamp makes more contacts with the entry DNA in hPol I, suggesting a more stabilized hPol I–DNA engagement during transcription elongation, in line with the transcription of more complex and longer rDNA substrate in human cells^29^. The flexible stalk in hPol I is similar to that of Pol II but differs from the relatively fixed stalk–clamp of yPol I, suggesting that the flexible stalk may accommodate binding of human-specific transcription factors, such as TBP-containing selectivity factor 1 (SL1) and upstream binding factor (UBF)^30–32^. The functional effect of these human-specific structural features (clamp and stalk) in hPol I requires further investigation.

### The catalytic center of Pol I EC in the pre- and post-translocation states

**Fig. 3:**
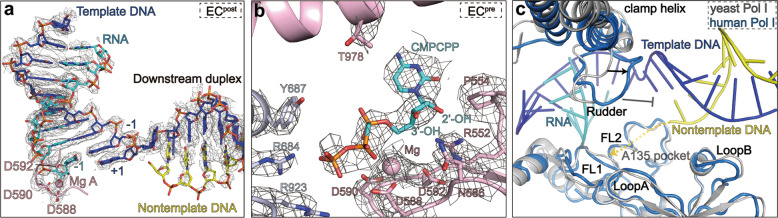
The catalytic center of Pol I in the post- and pre-translocation states. **a** Cryo-EM map and structural model of the DNA–RNA hybrid in the Pol I EC^post^. Critical residues involved in catalysis are shown in sticks. The magnesium cation in the metal A position is shown in pink ball. **b** Close-up view of the catalytic center of Pol I EC^pre^. The cryo-EM map is shown in mesh and critical residues are shown in sticks. **c** Conformational differences of hPol I (marine) and yPol I (gray) around the transcription fork. The yellow dash line indicates the putative path of the nontemplate strand.

The EC^post^ structure reveals characteristic DNA–RNA hybrid and catalytic center in the post-translocation state (Fig. 3a). The “metal A” magnesium cation is coordinated by three highly conserved aspartate residues (D592, D590, D588) of RPA1 and binds 3′ end of the growing RNA transcript. The nucleotides are mainly stabilized by subunits RPA1, RPA2, and RPABC1, and most of the nucleotide-binding residues are conserved across species^13,14^.

The cryo-EM map of the EC^pre^ shows well-resolved density of the CMPCPP at the +1 nucleotide addition site (Fig. 3b), similar to previously reported structures of yeast Pol I and Pol II in the pre-translocation state^33,34^. The phosphate group of the CMPCPP is stabilized by R684 and R923 of RPA2, two invariant residues in yeast and human Pol I. The conserved residues N586 and R552 of RPA1 are located near the 3′ and 2′ hydroxyl group of the CMPCPP, respectively, similar to that in yeast Pol I and Pol II structures^34,35^. Residue P554 of RPA1 is located close to the cytosine of CMPCPP. These interactions serve to recognize all types of NTP in the addition site. Residue T978 of RPA1 points toward the CMPCPP, and this conserved residue may participate in detecting the base pairing of +1 site in yPol II^36^. Residue Y687 of RPA2 around the CMPCPP is involved in proofreading (discussed below). The positioning of the NTP substrate in the active site is similar to that in yPol I structure^17^ (Supplementary Fig. S5b), indicating a highly conserved catalytic mechanism.

The rudder, fork loops 1/2, and loops A/B work together to stabilize the transcription fork and prevent re-association of the template and nontemplate strands (Fig. 3c). Compared to that of yPol I, the rudder (residues 403–416 of RPA1) in hPol I EC^post^/EC^pre^ is closer to the DNA–RNA scaffold due to the more closed clamp and may better stabilize the transcription fork. Other elements are similarly positioned in yPol I and hPol I.

### Structure of Pol I in the backtracked state reveals the post-cleavage conformation

RNA polymerases can move backwards on DNA template to remove the RNA 3′ end nucleotides from the active site when encountering transcription barriers, such as mismatched NTP addition^37^ and ultraviolet-induced damaged DNA^38^. It is known that transcription arrest occurs on the backtracked Pol II and reactivation of the arrested Pol II requires an additional transcription factor IIS (TFIIS), which cleaves the mismatched RNA^39^. The equivalent factors, RPA12 in Pol I and RPC11 in Pol III, are incorporated into Pol I and Pol III, respectively, and reorganize the active site and mediate cleavage of the mismatched RNA for proofreading^13,40^. In Pol I, the N-ribbon of RPA12 resembles that of the Pol II subunit Rpb9 and the C-ribbon resembles that of TFIIS^19^. Although the backtracking of Pol II has been reported^39,41,42^, the post-cleavage state was not observed in previous studies, in which the negatively-charged residues D and E of TFIIS have been mutated to deactivate its cleavage activity^39,42^. The mechanism of backtracking and RNA cleavage in Pol I-mediated transcription remains incompletely understood.

**Fig. 4:**
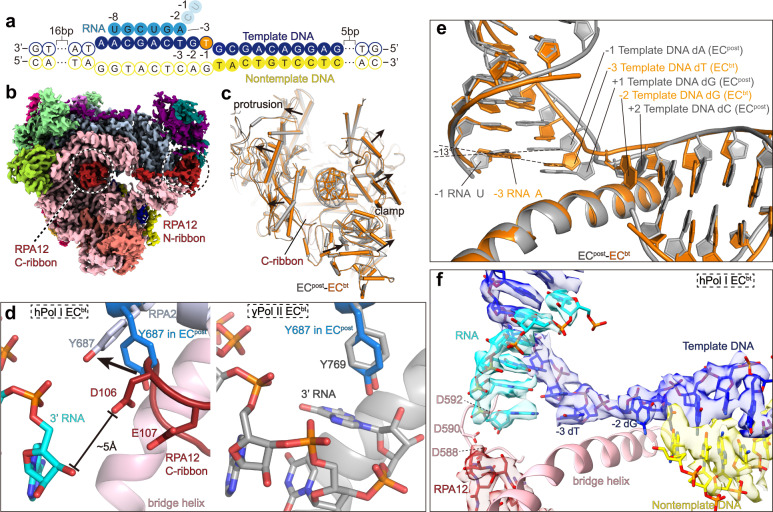
Structure of Pol I EC in the backtracked state. **a** Schematic diagram of the transcription scaffold used in assembly of Pol I EC^bt^. The dA^−1^ in template strand was changed to dT^−1^, generating mismatched base pair of DNA–RNA hybrid. The U^−1^ and C^−^^2^ of the RNA were cleaved in the assembled EC^bt^. **b** Cryo-EM map of EC^bt^ shows that the C-ribbon of RPA12 is inserted into the active site. **c** Structure comparison shows that the cleft of EC^bt^ (orange) is wider than that of EC^post^ (gray). **d** Left panel shows the close-up view of the tip of RPA12 C-ribbon in the active site and its interaction with the gating tyrosine and 3′ end RNA. Residue Y687 in EC^post^ is shown in sticks and colored in marine. Conformational difference in residue Y687 in the two states is indicated with black arrow. Right panel shows the same view of yPol II EC^bt^ without TFIIS (gray; PDB: 3GTJ)^42^. Residue Y687 in hPol I EC^post^ is shown (marine) for comparison. **e** Comparison of the DNA–RNA hybrid in hPol I EC^bt^ (orange) and EC^post^ (gray). **f** Cryo-EM map and structural model of catalytic center of the Pol I EC^bt^. Cryo-EM map is shown in transparent surface.

To obtain the structure of Pol I EC^bt^, we assembled the complex with the nucleotide at the −1 site of the template DNA converted from dA to dT, generating a dT–U mismatch (Fig. 4a). Consistent with the cleavage of dinucleotide in the in vitro transcription assay (Supplementary Fig. S1b, lanes 5–9), the remaining 6-nt RNA was evidently observed in the cryo-EM map (Fig. 4f). The metal A was invisible at the original position near the three aspartic acids (Supplementary Fig. S4d). The structure of EC^bt^ represents Pol I in the backtracked state after cleavage of the mismatched RNA.

The C-ribbon of RPA12 was not observed in the EC^pre^ and showed very weak density in the EC^post^. In contrast, the cryo-EM of EC^bt^ reveals well-ordered C-ribbon of RPA12 within the funnel (Fig. 4b). Consistently, the bridge helix is slightly extended (Supplementary Fig. S4c) and the funnel and the cleft of EC^bt^ are slightly wider than those of EC^post^ to permit the entry of RPA12 C-ribbon (Fig. 4c), which may otherwise clash with the rim of the funnel in the EC^post^. A conserved tyrosine located in the active site is called “gating tyrosine,” which can block backward movement of RNA. In yPol II EC^bt^ without TFIIS, the “gating tyrosine” Y769 of Rpb2 clearly blocks the backtracked RNA^42^. In hPol I EC^bt^, Y687 is “opened” by the C-ribbon in EC^bt^ and permits the backward translocation of 3′ RNA from the active site (Fig. 4d and Supplementary Fig. S4d). The tip residues D106 and E107 of the C-ribbon are in close proximity to the bridge helix and are ~5 Å away from the 3′ end of the modeled RNA (Supplementary Fig. S4e). These residues are invariant in yeast and human Pol I and may coordinate the nucleophilic water and magnesium cation to cleave the phosphodiester bond. The arrangement of this RPA12 tip is consistent with the previously proposed S_N_2 mechanism to cleave the scissile phosphodiester bond in Pol II^39^. While it has been proposed that metal B is important for the cleavage activity^39^, metal A was observed in EC^pre^/EC^post^ but not in EC^bt^ (Supplementary Fig. S4d), suggesting that metal A may also be involved in RNA cleavage.

Compared to EC^post^, the template strand in the EC^bt^ has obvious positional shift (Fig. 4e). For example, the dT^−3^ of the EC^post^ moves to the −1 site in EC^bt^, which is located between the positions −1 and +1 in the EC^post^. This base tilts by ~13° to generate base pair with the first RNA base at the −1 site in EC^bt^. The dG^−2^ of the EC^post^ moves over the bridge helix and is positioned to the +1 site in the EC^bt^ and this unpaired base points toward the downstream DNA duplex. The catalytic center and the DNA–RNA hybrid position of Pol I EC^bt^ are generally similar to that of the reactivation intermediate Pol II^39^ (Supplementary Fig. S5d), in which the mutated TFIIS resulted in a similar rotation of the gating tyrosine, whereas the long backtracked RNA was not cleaved. In contrast, no DNA–RNA hybrid rearrangement was observed in the structure of Pol II EC containing mutated TFIIS and a short-mismatched RNA^42^. Distinct from the above observations in Pol II, Pol I leads to rearrangement of the catalytic center and cleaves the substrate containing a short-mismatched RNA in the presence of active RPA12.

### Disease-associated mutations of the hPol I

**Fig. 5:**
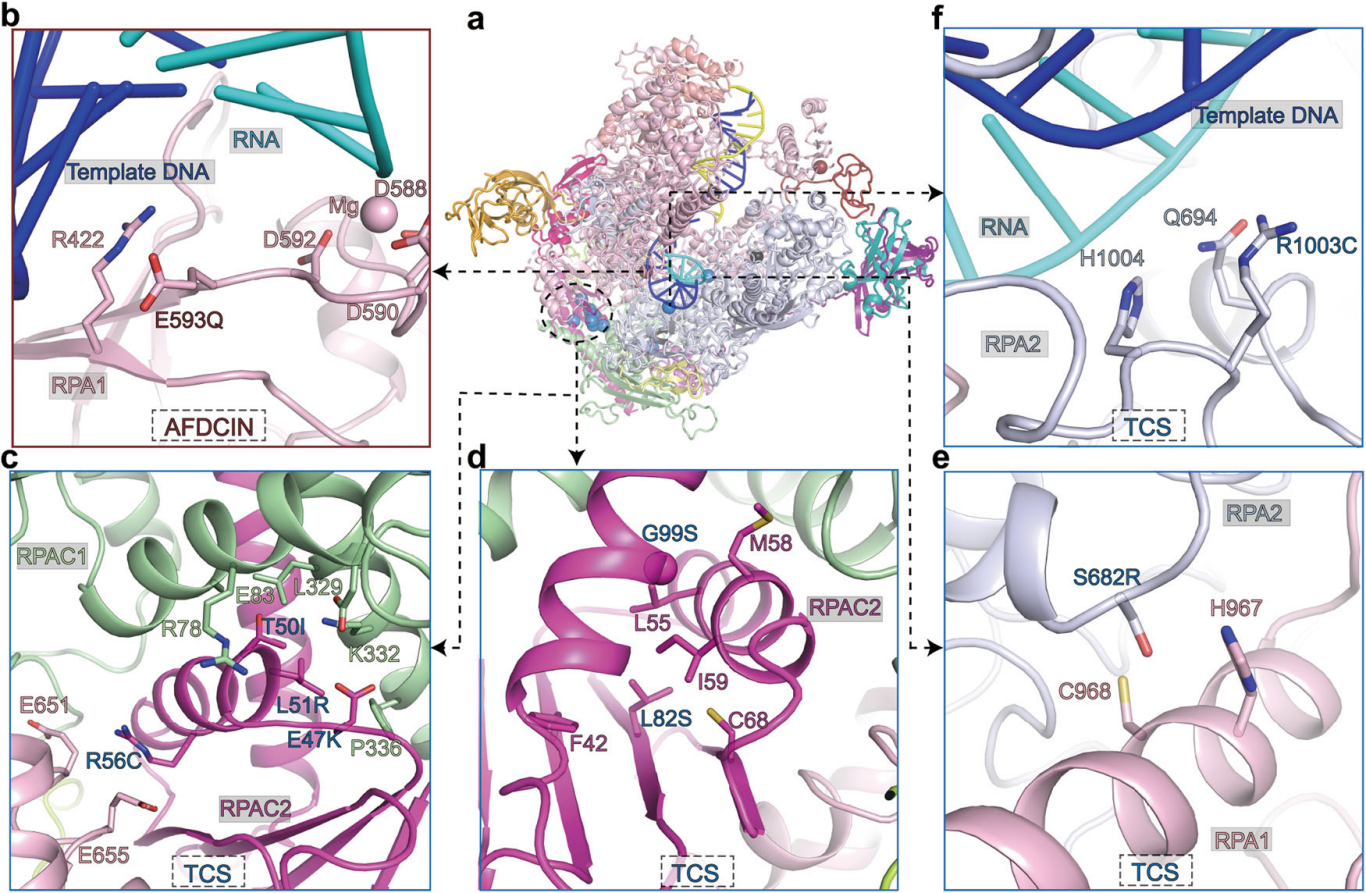
Disease-associated mutations in hPol I. **a** Overall structure of the hPol I EC^post^ with spheres indicating the positions of mutations associated with AFDCIN (red), neurodegenerative diseases (black), and TCS (blue). **b**–**f** Close-up views of the positions of disease-associated mutations. Mutant residues and potential contacting residues are shown in sticks.

The hPol I-mediated transcription is critical for ribosome production, regulation of cell growth, and proliferation. Mutations of Pol I subunits result in perturbation of ribosome biogenesis during development and lead to ribosomopathies, such as severe neurodegenerative diseases, acrofacial dysostosis-type Cincinnati (AFDCIN) and Treacher Collins Syndrome (TCS)^43–47^ (Fig. 5a).

The mutation E593Q near the active site of RPA1 causes AFDCIN, a cranioskeletal malformation syndrome^43^. A recent study shows that E593Q-containing Pol I stably binds rDNA chromatin and inhibits wild-type Pol I condensate formation, leading to repression of rRNA synthesis^48^. Notably, residue E593 is positioned near the conserved magnesium-binding aspartate residues (D592, D590, and D588) (Fig. 5b). The replacement of E to Q may affect the geometry of the magnesium coordination and nucleotide addition, thus decreasing Pol I transcription and generating a dominate-negative polymerase.

Mutations E47K, T50I, L51R, R56C, L82S, and G99S in the N-terminal helixes of RPAC2 are associated with TCS^44,45^ (Fig. 5c, d). These residues involve intermolecular interactions between RPAC1 and RPAC2 and the above mutations may lead to the destabilization of RPAC1–RPAC2 heterodimer and affect Pol I activity. Moreover, residue R56 of RPAC2 binds residues E651 and E655 of RPA1 in Pol I but does not generate similar charge–charge interaction in Pol III (Fig. 5c and Supplementary Fig. S5e). As a shared subunit of Pol I and Pol III, the deletion of RPAC2 in zebrafish impaired Pol I-supported transcription of 47S RNA but had no effect on Pol III-supported transcription of 5S RNA^49^. Thus, dysfunction of Pol I may play a major role in RPAC2-related TCS. Moreover, TCS can also result from RPA2 subunit mutations. Mutation S682R of RPA2 may destabilize the bridge helix of RPA1 (Fig. 5e); mutation R1003C of RPA2 is located at the DNA–RNA hybrid-binding region and may affect nucleotide association^46^ (Fig. 5f). These mutations may decrease Pol I activity and thus affect ribosome biogenesis during development.

## Discussion

In this study, we reconstituted human Pol I complex and determined the cryo-EM structures of Pol I EC in the pre-translocation, post-translocation, and backtracked states. The overall hPol I EC structures reveal a more closed DNA-binding clamp and a flexible one-subunit stalk. The Pol I in the post-cleavage backtracked state has wider cleft and RPA12 in the funnel may favor RNA cleavage upon backtracking of the mismatched RNA. Structural comparison reveals structural differences between human Pol I and other polymerases, which may reflect their functional differences. Our structures suggest potential implication of disease-associated mutations of hPol I and provides basis for further studies of hPol I transcription initiation.

In human cells, the total number of rDNA repeats was estimated to be in the range of ~400 copies, and each rDNA repeat (~43 kb) consists of regulatory elements within an intergenic spacer (IGS) of ~30 kb and the 47S pre-rRNA coding region of ~13 kb^4,50^, while in *Saccharomyces cerevisiae*, each rDNA repeat (~9.1 kb) consists of ~6.6 kb 35S coding region and a short IGS^29,51^. The *S. cerevisiae* and mammalian Pol I elongate at an average speed of 60 nt/s and 91 nt/s in cells, respectively^52,53^. The difference in transcription speed may be related to the observed structural differences. Compared to yPol I, hPol I has a more closed DNA-binding clamp, which may generate more stable binding of the rDNA during transcription elongation and support high transcription processivity.

It is known that RPA43 is involved in transcription initiation^54^. In all our structures, the stalk subunit RPA43 is flexible relative to the core module, distinct from the stably associated stalk in yPol I. The compositional and conformational differences in the yPol I stalk and hPol I stalk may accommodate the differences in transcription initiation in yeast and human systems. In recently reported structures of yPol I initiation complexes^35,55^, Rrn3 binds A43 and participates in the activation of Pol I transcription initiation, while Pol I core module interacts with the core factor (CF) formed by Rrn6, Rrn7, and Rrn11. The yPol I-mediated transcription initiation also requires TATA box-binding protein (TBP), Hmo1 and upstream activation factor^32,56^, which have not been structurally determined. The hPol I-mediated transcription initiation involves UBF, RRN3, and SL1 formed by TBP, TAF1A, TAF1B, TAF1C, TAF1D, and TAF12^30,31,57–61^. The yeast Rrn3 and human RRN3 share high sequence similarity, suggesting similar binding of Rrn3/RRN3 to A43/RPA43 in the yPol I and hPol I. The yeast CF subunits Rrn6, Rrn7, and Rrn11 are distantly related to human SL1 subunits TAF1C, TAF1B, and TAF1A, respectively^62^. However, the other two SL1 subunits, TAF1D and TAF12, do not exist in the yPol I system.

The mechanistic studies of polymerase proofreading have been mainly focused on Pol II. When Pol II encounters mismatched DNA–RNA, RNA fraying occurs and Pol II pauses and backtracks by 1 bp. However, further backtracking is hindered by the gating tyrosine. Transcription elongation continues if the mismatched RNA is cleaved by intrinsic cleavage activity of the polymerase^39,42^. However, if the mismatched RNA backtracks beyond the gating tyrosine at some occasions, transcription arrest occurs and TFIIS is required to bind to reactivate the arrested Pol II. Our study suggests a mechanism of coordinated backtracking and RNA cleavage in Pol I-mediated transcription. The mismatched DNA–RNA possibly facilitates the Pol I to open the cleft and funnel to permit the entry of RPA12, which pushes the gating tyrosine Y687 aside to open the gate and allows the mismatched RNA to pass through. The catalytic center rearranges the nucleotides and exposes the scissile phosphodiester bond between nucleotide −1 and +1 to the negatively-charged RPA12 tip, which may facilitate the cleavage of the phosphodiester bond. Reactivation and continued transcription may occur upon the addition of next NTP. These evidences are consistent with previous report that yeast Pol I transcribes faster than Pol II and pauses less often, and Pol I is more efficient in backtracking recovery than Pol II^15^.

The hPol I EC that we assembled with the non-mismatched scaffold for extension assay generated laddered bands (Supplementary Fig. S1b, lane 1) as previously observed in yPol I^18^, indicative of cleaved RNA by automatically backtracked Pol I. This is consistent with a recent study that the stable structure of nascent RNA favors forward nucleotide addition of Pol I and prohibits backtracking^63^, indicating that Pol I transcription backtracking may be further regulated by transcription-coupled events.

## Materials and methods

### Protein expression and purification

The open reading frames of 13 subunits of hPol I were individually subcloned into a modified pCAG vector^64^. PAF53 is the only subunit that is N-terminally Protein A tagged and all the rest of subunits are untagged. Except that RPA1 and RPA2 are individually cloned, the expression cassettes of the other 11 subunits were merged into 5 plasmids (in particular, tagged PAF53 and PAF49 were merged as the first plasmid; RPA43 and RPA12 were merged as the second plasmid; RPAC1 and RPAC2 were merged as the third plasmid; RPABC1 and RPABC2 were merged as the fourth plasmid; RPABC3, RPABC4, and RPABC5 were merged as the fifth plasmid) and all plasmids are co-transfected into Expi293F suspension cells using PEI. After being cultured at 37 °C for 72 h, cells were harvested and lysed in lysis buffer (50 mM HEPES, pH 7.4, 300 mM NaCl, 0.25% CHAPS, 10 μM ZnCl_2_, 5 mM ATP, 5 mM MgCl_2_, 10% glycerol, 2 mM DTT, 1 mM PMSF, 1 μg/mL Benzamidine, 1 μg/mL Pepstatin, and 1 μg/mL Leupeptin) at 4 °C. The supernatant was incubated with IgG-agarose beads (Smart Lifesciences) at 4 °C for 3 h, and beads were extensively washed with wash buffer (50 mM HEPES, pH 7.4, 300 mM NaCl, 0.1% CHAPS, 10% glycerol, 5 mM ATP, 5 mM MgCl_2_, 10 μM ZnCl_2_ and 2 mM DTT). Protein was digested using Ulp1 protease overnight to remove tags, and the complex was eluted with elution buffer (50 mM HEPES, pH 7.4, 300 mM NaCl, 0.1% CHAPS, 10% glycerol, 2 mM MgCl_2_, 10 μM ZnCl_2_, and 2 mM DTT). The eluted protein was diluted to 100 mM NaCl and loaded on Mono Q (5/50 GL, GE Healthcare). The bound protein was eluted with increasing concentrations of NaCl from 0.1 M to 1 M and Pol I complex was eluted at 360 mM NaCl. Fractions containing Pol I complex were pooled, concentrated using a 100-kDa cut-off centrifugation filter unit (Millipore) to ~2 mg/mL, and then dialyzed against low-salt buffer (similar to Mono Q buffer but containing 150 mM NaCl) overnight. The complex was then flash-frozen in liquid nitrogen and stored at −80 °C.

### Complex assembly

For the preparation of EC^post^, Pol I was incubated with a 46-bp transcription scaffold containing an 11-nt mismatched bubble and an 8-nt RNA (Supplementary Table S2). The oligonucleotides were dissolved in DEPC H_2_O to a final concentration of 100 mM, mixed in equimolar concentration, heated to 95 °C for 5 min, and cooled to 20 °C at a rate of 1 °C/min. To obtain DNA–RNA hybrid, DNA duplex was incubated with a 1.2-fold molar of RNA for 5 min at 45 °C and then gradually cooled to 4 °C. Ten μL Pol I was incubated with a 1.5-fold molar excess of DNA–RNA scaffold for 10 min at 25 °C. Sample was dialyzed against dialysis buffer (25 mM HEPES, pH 7.4, 150 mM NaCl, 2 mM MgCl_2_, 2 mM DTT) at 4 °C using Slide-a-lyzer mini dialysis device (10,000 molecular weight cut-off, Thermo Fisher). The EC^pre^ and EC^bt^ were assembled similarly but with a few modifications. The EC^pre^ was assembled by incubating the EC^post^ with additional 1 mM CMPCPP. The EC^bt^ was assembled uniformly as EC^post^, except with −1 site mismatched template DNA (Supplementary Table S2).

### Transcription assay

Four picomoles of polymerase was incubated for 30 min at 20 °C with 4 pmol pre-annealed minimal nucleic acid scaffold (Supplementary Table S2). For RNA elongation, complexes were incubated in the presence of 1 mM NTPs at 28 °C for 20 min in transcription buffer (30 mM HEPES, pH 7.5, 100 mM NaCl, 5 mM MgCl_2_, 10 μM ZnCl_2_, 10% glycerol, and 2 mM DTT). Reactions were stopped by addition of an equal volume of 2× loading buffer (8 M urea, 2× TBE) at different times and incubation for 5 min at 95 °C. The FAM-labeled RNA extension products were separated by denaturing gel electrophoresis (0.5 pmol RNA per lane) and visualized with Tanon 4600SF. For RNA cleavage assays, Pol I was incubated with pre-annealed backtrack-scaffold (Supplementary Table S2) at 16 °C, and reactions were stopped at different times and analyzed by gel electrophoresis as above.

### Cryo-EM sample preparation

For negative staining EM grid preparation, samples (5 µL at a concentration of ~0.035 mg/mL) were applied onto glow-discharged copper grids supported by a continuous thin layer of carbon film for 60 s before being negatively stained by 2% (w/v) uranyl formate solution at room temperature. The grids were prepared in the Ar/O_2_ mixture for 15 s using a Gatan 950 Solarus plasma cleaning system with a power of 35 W. The negatively stained grids were loaded onto a Thermo Fisher Scientific Talos L120C microscope equipped with a Ceta CCD camera and operated at 120 kV at a nominal magnification of 92,000×, corresponding to a pixel size of 1.58 Å on the specimen.

For cryo-EM grid preparation, samples (4 μL at a concentration of ~1.5 mg/mL) were applied to freshly glow-discharged Quantifoil R1.2/1.3 holey gold grids. After incubation for 5 s at 4 °C and 100% humidity, the grids were blotted for 8.5 s with force 13 in a Thermo Fisher Scientific Vitrobot Mark IV and plunge-frozen in liquid ethane at liquid nitrogen temperature. The grids were prepared in the H_2_/O_2_ mixture for 20 s using a Gatan 950 Solarus plasma cleaning system with a power of 5 W. The ø 55/20 mm blotting paper (TED PELLA) was used for plunge freezing.

### Data collection

The cryo-EM grids of Pol I EC were loaded onto a Thermo Fisher Scientific Titan Krios transmission electron microscope and operated at 300 kV for data collection. All the cryo-EM images were automatically recorded by a Gatan K2 Summit direct electron detector in the super-resolution counting mode using Serial-EM^65^ with a nominal magnification of 130,000× in the NPTEM mode, which yielded a super-resolution pixel size of 0.527 Å on the image plane, and with a defocus value ranging from 1.5 μm to 2.5 μm. Each micrograph stack was dose-fractionated to 32 frames with a total electron dose of ~50 e^–^/Å^2^ ^66^ and a total exposure time of 6.94 s. For the dataset of Pol I EC^pre^, EC^post^, and EC^bt^ samples, 3283, 2074, and 2854 micrographs were collected for further processing, respectively.

The cryo-EM grids of EC^post^-crosslinking sample were loaded onto a Thermo Fisher Scientific Arctica transmission electron microscope and operated at 200 kV for data collection. All the cryo-EM images were automatically recorded by a Gatan K3 Summit direct electron detector in the super-resolution counting mode using Serial-EM with a nominal magnification of 36,000× in the TEM mode, which yielded a super-resolution pixel size of 0.55 Å on the image plane, and with a defocus value ranging from 1.5 μm to 2.5 μm. Each micrograph stack was dose-fractionated to 40 frames with a total electron dose of ~50 e^–^/Å^2^ and a total exposure time of 3.009 s. For the dataset of EC^post^-crosslinking sample, 505 micrographs were collected for further processing.

### Image processing

For cryo-EM data, drift and beam-induced motion corrections were applied on the super-resolution movie stacks using MotionCor2^67^ and binned 2-fold to a calibrated pixel size of 1.054 Å/pix. The defocus values were estimated by Gctf^66^ from summed images without dose weighting. Other procedures of cryo-EM data processing were performed with RELION v3.0^68,69^ and cryoSPARC v2^68,69^ using the dose-weighted micrographs.

For the datasets of the Pol I EC^pre^, 1,141,229 particles were picked by automatic particle picking in RELION without reference and subjected to reference-free two-dimensional (2D) classification. In all, 946,031 particles were selected from good 2D classes for three-dimensional (3D) classification in RELION. A total of 382,890 particles were selected from good 3D classes, which were used for the heterogeneous refinement in cryoSPARC and CTF, yielding a reconstruction of Pol I EC^pre^ at 2.89 Å resolution.

For the datasets of the Pol I EC^post^, 618,806 particles were picked by automatic particle picking in RELION without reference and subjected to reference-free 2D classification. In all, 389,117 particles were selected from good 2D classes for 3D classification in RELION. A total of 282,280 particles were selected from good 3D classes, which were used for the heterogeneous refinement in cryoSPARC and CTF, yielding a reconstruction of Pol I EC^post^ at 2.81 Å resolution.

For the datasets of the Pol I EC^bt^, 676,465 particles were picked by automatic particle picking in RELION without reference and subjected to reference-free 2D classification. In all, 581,698 particles were selected from good 2D classes for 3D classification in RELION. A total of 152,653 particles were selected from good 3D classes, which were used for the heterogeneous refinement in cryoSPARC and CTF, yielding a reconstruction of Pol I EC^bt^ at 3.01 Å resolution.

For the datasets of the EC^post^ (crosslinking) complex, 353,482 particles were picked by automatic particle picking in RELION without reference and subjected to reference-free 2D classification. In all, 250,226 particles were selected from good 2D classes for 3D classification in RELION. A total of 127,587 particles were selected from good 3D classes, which were used for the heterogeneous refinement in cryoSPARC and CTF, yielding a reconstruction of EC^post^ at 3.89 Å resolution.

All reported resolutions are based on the gold-standard (GS) Fourier shell correlation (FSC) = 0.143 criterion. The GSFSC curves were corrected for the effects of a soft mask with high-resolution noise substitution. All cryo-EM maps were sharpened by applying a negative *B*-factor estimation in cryoSPARC Sharpening Tools. All the visualization and evaluation of the 3D volume map were performed with UCSF Chimera or UCSF ChimeraX^70^, and the local resolution variations were calculated using cryoSPARC.

### Model building and structure refinement

The cryo-EM maps of the Pol I ECs were used for model fitting. The structures of yeast Pol I EC (PDB: 5M3F)^16^ was used as initial structural template, which was docked into the cryo-EM maps by rigid-body fitting using UCSF Chimera^70^ with the aid of AlphaFold prediction^20^. The structural models were built in COOT^71^ and refined in real space using Phenix^72^ with secondary structure and geometry restraints using the cryo-EM map of the Pol I EC. Overfitting of the model was monitored by refining the model in one of the two half maps from the gold-standard refinement approach and testing the refined model against the other map^73^. Statistics of the map reconstruction and model refinement can be found in Supplementary Table S1. The final models were evaluated using MolProbity^74^. Maps and model representations in the figures were prepared by PyMOL (https://pymol.org/)^75^, UCSF Chimera, or UCSF ChimeraX^76^.

## Supplementary information

Supplementary information

Supplementary Video S1

Supplementary Video S2

## Data Availability

The cryo-EM maps have been deposited in the EM Databank under accession numbers: EMDB-31877 (EC^post^), EMDB-31876 (EC^pre^), and EMDB-31878 (EC^bt^). Atomic coordinates have been deposited in the Protein Data Bank with PDB IDs: 7VBB (EC^post^), 7VBA (EC^pre^), and 7VBC (EC^bt^).
