# Supplementary material for: Structure of the human RNA polymerase I elongation complex
Source: Cell Discov. 2021 Oct 20;7:97. doi: 10.1038/s41421-021-00335-5 (PMC8528822; doi:10.1038/s41421-021-00335-5)
Supplement: Supplementary file 1 — Supplementary information [file 41421_2021_335_MOESM1_ESM.pdf]

Supplementary information

Structure of the human RNA Polymerase I Elongation Complex

Dan Zhao[1#], Weida Liu[1#], Ke Chen[1#], Zihan Wu[1], Huirong Yang[1]*, and Yanhui Xu[1,2,3,4]*

[1]Fudan University Shanghai Cancer Center, Institutes of Biomedical Sciences, State Key Laboratory of Genetic Engineering, Shanghai Key Laboratory of Radiation Oncology, and Shanghai Key Laboratory of Medical Epigenetics, Shanghai Medical College of Fudan University, Shanghai 200032, China.

[2]The International Co-laboratory of Medical Epigenetics and Metabolism, Ministry of Science and Technology, China, Department of Systems Biology for Medicine, School of Basic Medical Sciences, Shanghai Medical College of Fudan University, Shanghai 200032, China.

[3]Human Phenome Institute, Collaborative Innovation Center of Genetics and Development, School of Life Sciences, Fudan University, Shanghai 200433, China

[4]State Key Laboratory of Reproductive Regulation and Breeding of Grassland Livestock School of Life Sciences, Inner Mongolia University, Hohhot, 010070, China

[#] These authors contributed equally to this work.

*To whom correspondence should be addressed. E-mail: yanghr@fudan.edu.cn, xuyh@fudan.edu.cn

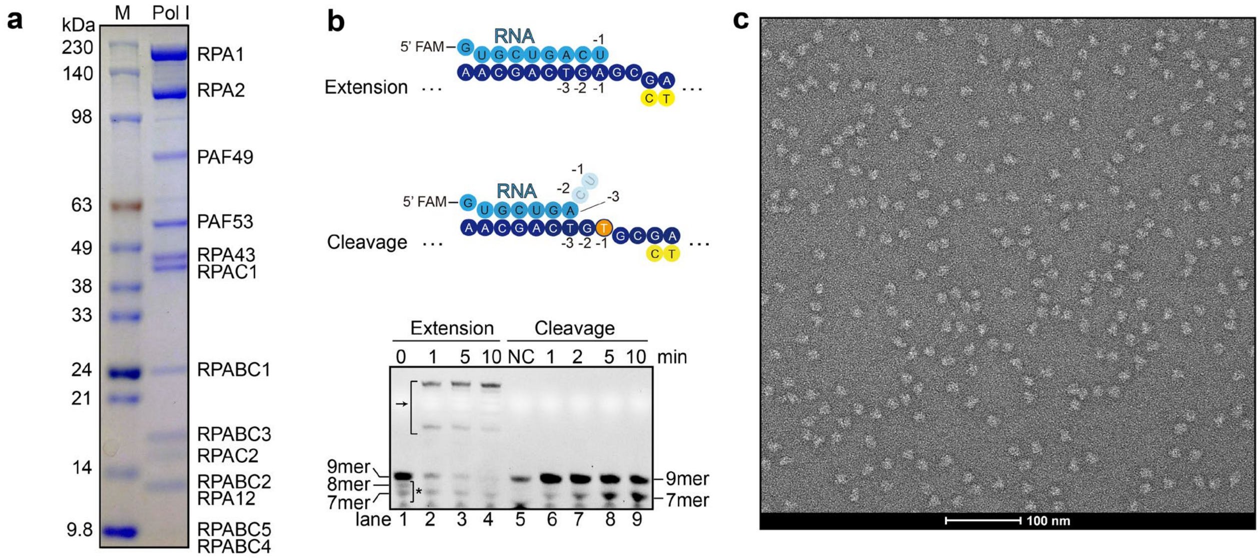

Supplementary Fig. S1 Sample preparation and transcription assay of the human Pol I

(a) The purified hPol I consists of all 13 subunits. The complex was subjected to SDS-PAGE followed by Coomassie blue staining. **(b)** RNA extension and cleavage assay of the purified hPol I shows robust activity. Schematic model of DNA-RNA substrates (only the critical regions shown) used in the assay is shown as in Fig. 1a and Fig. 4a. The translucent nucleotides of the cleavage sequence represent the two nucleotides cleaved in the assay. In the bottom panel, the length of 5'-FAM RNA is labelled. The arrow indicates extension products. The asterisk indicates the RNA products from intrinsic RNA cleavage activity of Pol I. The negative control (NC) of the cleavage assay was performed by adding 5'-FAM-RNA without DNA scaffold to the Pol I at the same condition. **(c)** The typical negative stain micrograph of apo hPol I shows homogenous monomeric polymerase complex.

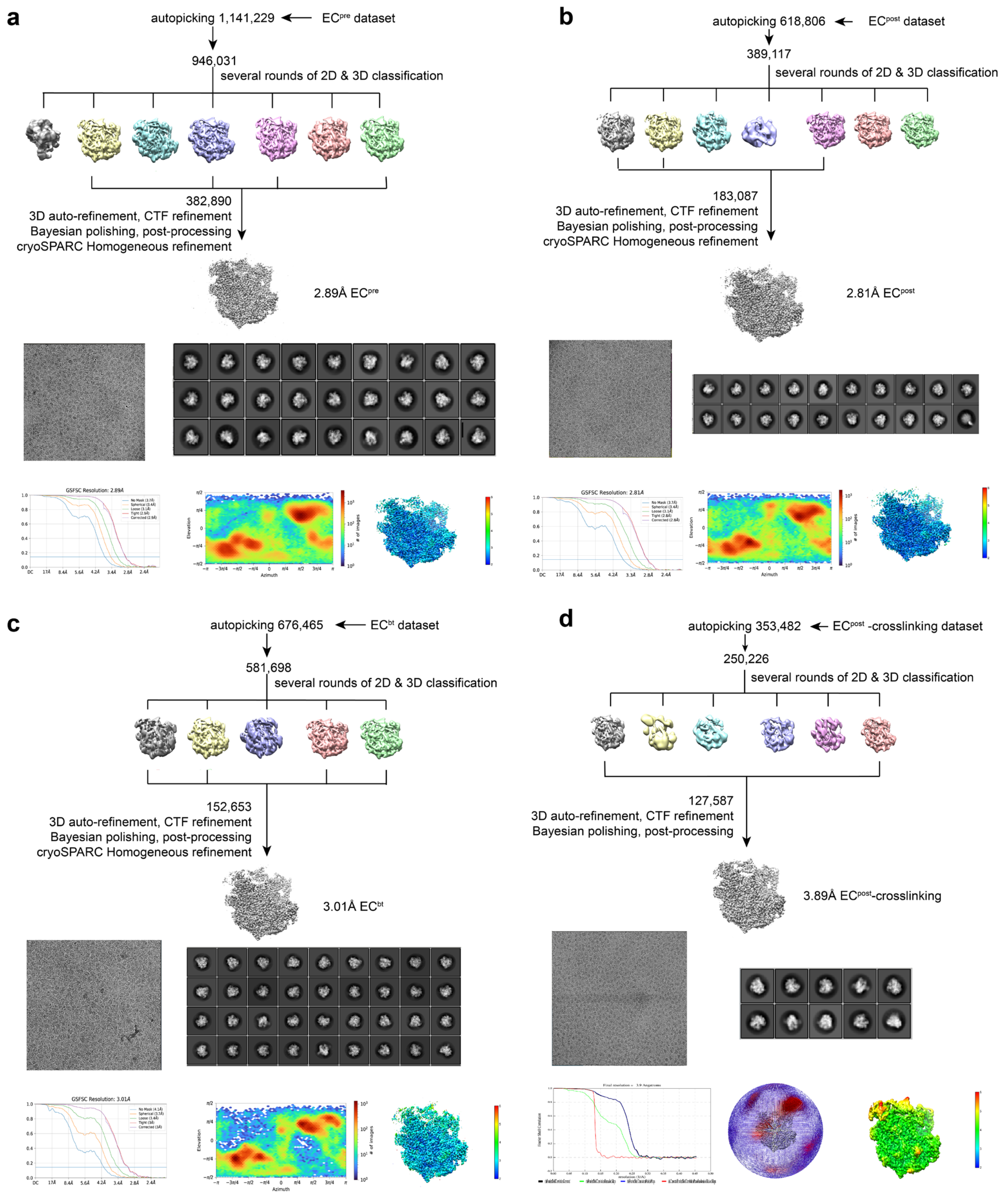

Supplementary Fig. S2 Data collection and image processing.

Cryo-EM reconstructions of Pol I EC^{pre} (a), EC^{post} (b), EC^{bt} (c) and EC^{post}-crosslinking (d) accordingly. In each figure, the top panel is the flow-chart of the cryo-EM image processing; the

middle panel shows the representative cryo-EM raw micrograph and 2D classification; the bottom left panel is the FSC curve of the corresponding map; the bottom middle and right panels are the local resolution estimation and orientation of the cryo-EM reconstructions of the corresponding map.

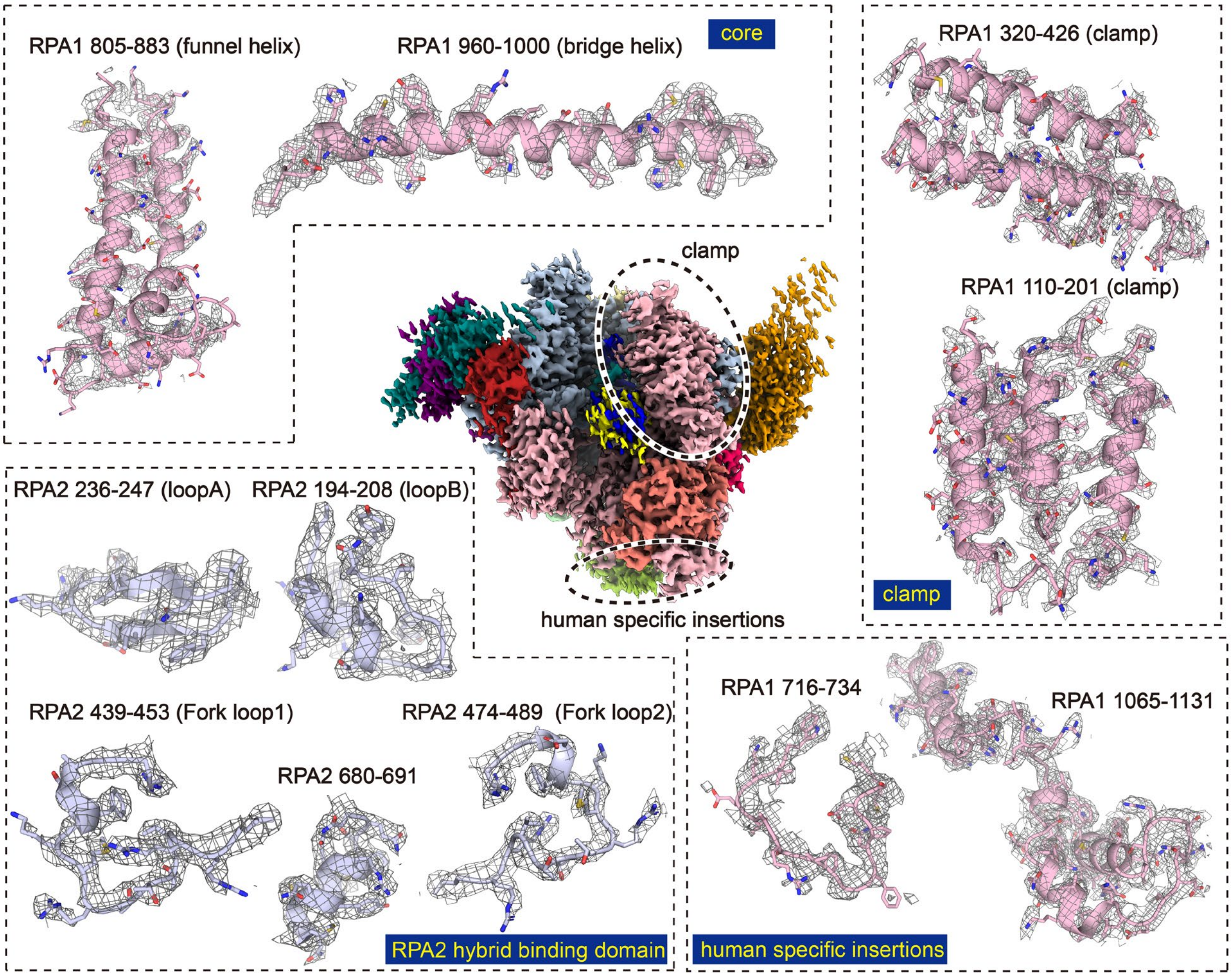

Supplementary Fig. S3 Cryo-EM map and structural model of hPol I EC^{post}.

Overall cryo-EM map of hPol I EC^{post} is shown in the center. The cryo-EM maps of representative regions are shown in mesh and structural models shown in cartoon. Most of the side chains fit in corresponding density, indicating the structure was correctly built.

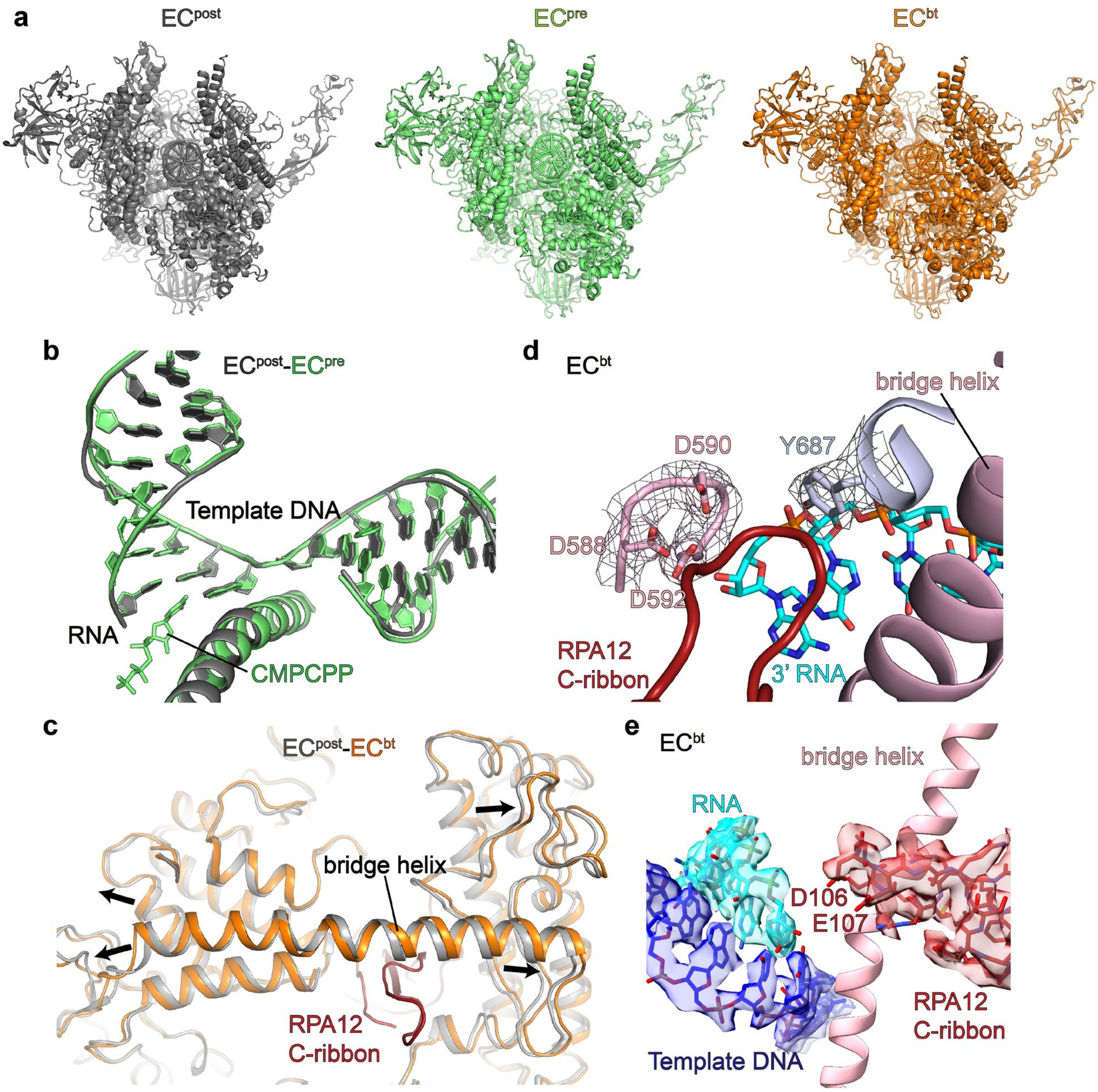

Supplementary Fig. S4 Structural comparison of human Pol I EC in the three states.

(a) The structural models of human EC^{post} (gray), EC^{pre} (green) and EC^{bt} (orange). **(b)** Comparison of the DNA-RNA hybrid in the EC^{post} (gray) and EC^{pre} (green). **(c)** Comparison of the bridge helix and surround regions in the EC^{post} and EC^{bt}. The EC^{post} is colored in gray and EC^{bt} is colored in orange expect for the RPA12 C-ribbon (red). Structural differences are indicated with arrows. **(d-e)** Close-up views of the C-ribbon of RPA12 in the active site. The cryo-EM map is shown in mesh (d)

and transparent surface (e), respectively. Most of residues are well-fit into the cryo-EM map.

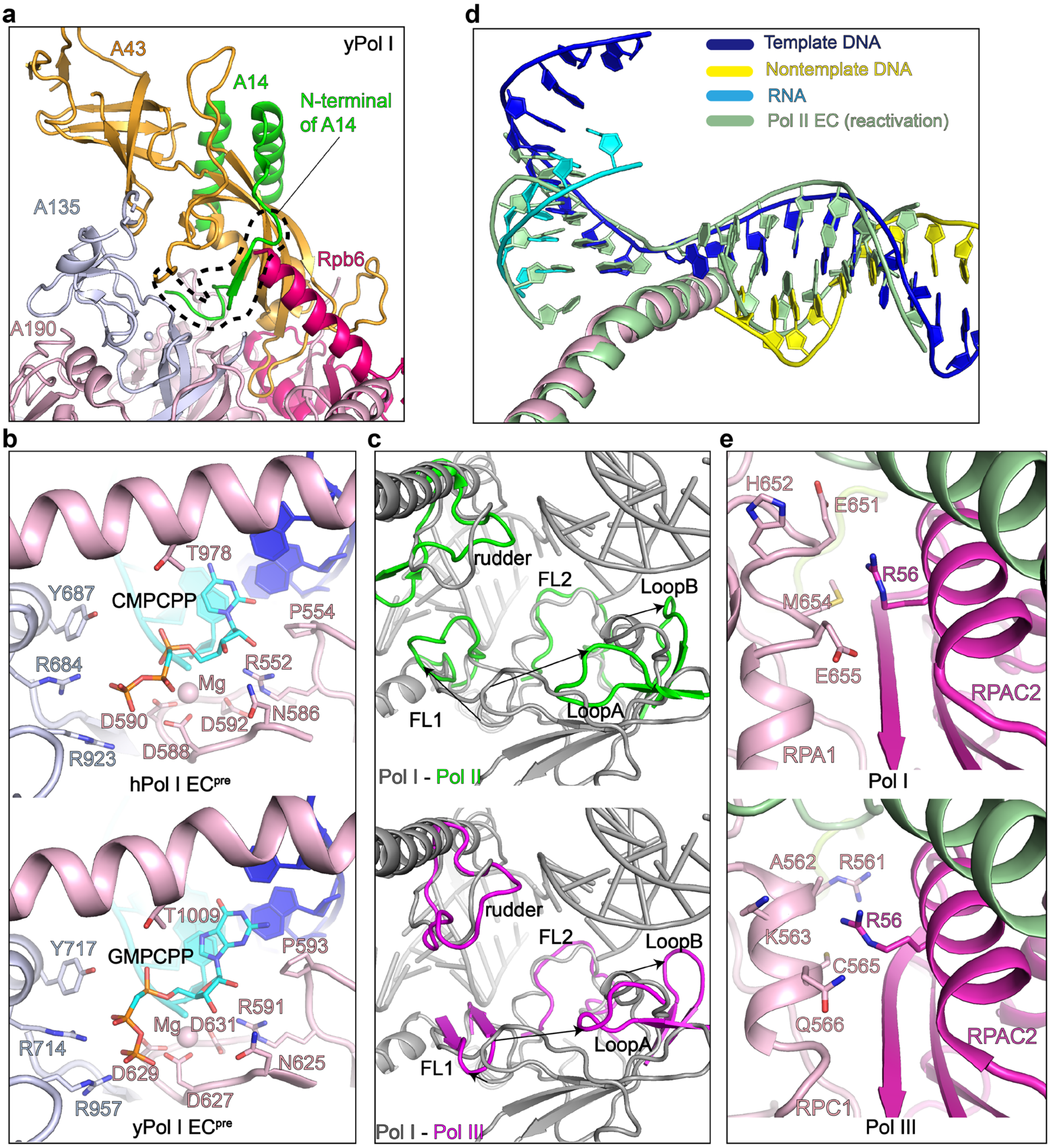

Supplementary Fig. S5 Structural comparison of hPol I with other RNA polymerases.

(a) Structural model of the stalk of yPol I (PDB: 5M3F)[1]. A14 is colored in green and other subunits are colored as in hPol I in Fig. 1. **(b)** The catalytic centers are similar in hPol I EC^{pre} and yPol I EC^{pre} (PDB: 6HKO)[2]. Subunits are colored as in Fig. 1 **(c)** The FL1, FL2, loopA, and loopB in the active site of hPol I form a narrow tunnel that directs the exit of the nontemplate DNA strand. Pol II (green;

PDB: 5FLM)[3] and Pol III (purple; PDB: 7D58)[4] show distinct conformations of these elements. **(d)** Comparison of DNA-RNA hybrid in hPol I EC^{bt} in the post-cleavage state and yPol II in the reactivation state (PDB: 3PO3)[5] with color diagram listed. **(e)** The conserved residue R56 of RPAC2 in Pol I and Pol III (PDB: 7D58)[4] makes distinct contact with RPA1/RPC1 in two enzymes.

a

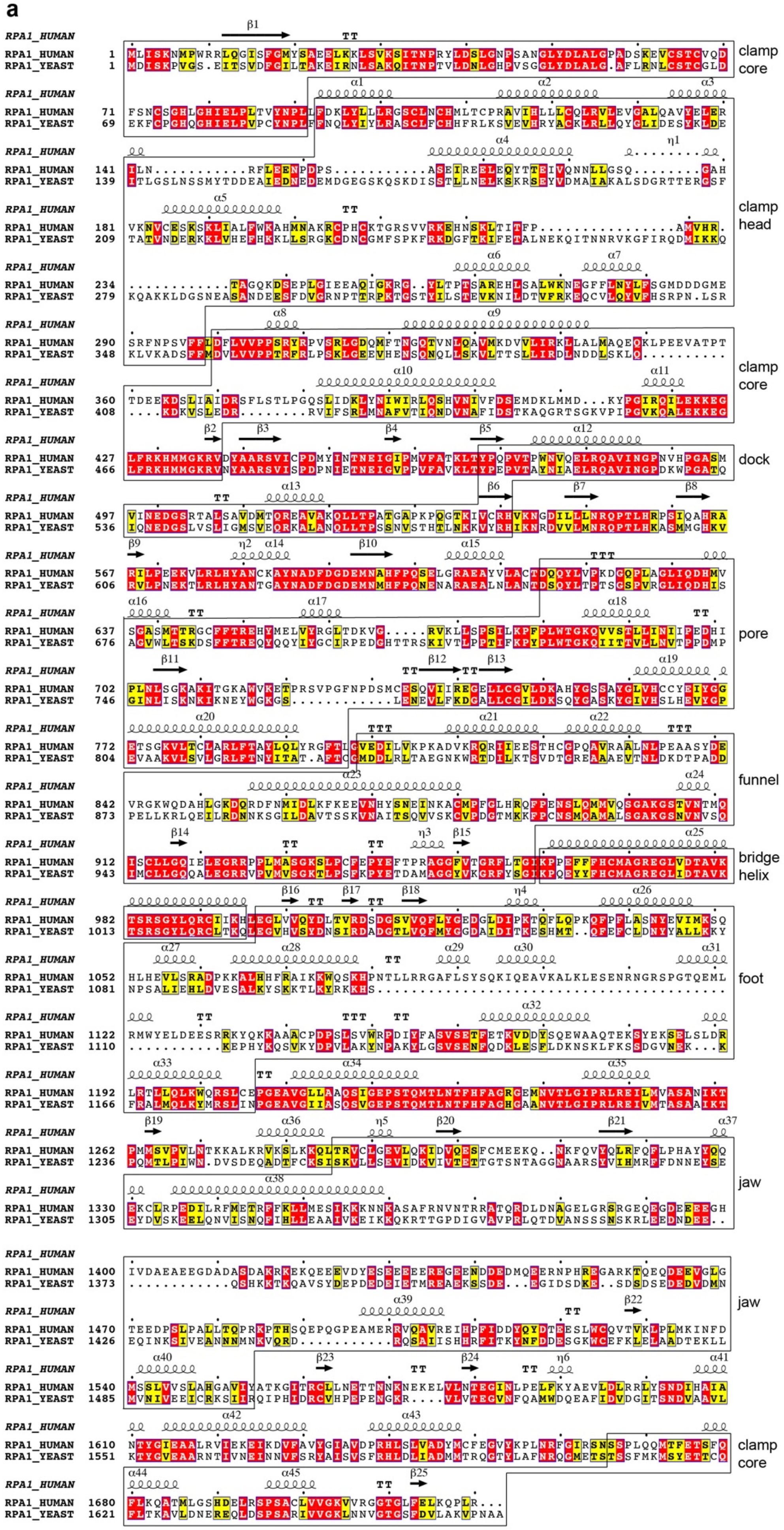
clamp core
clamp head
clamp core
dock
pore
funnel
bridge helix
foot
jaw
jaw
clamp core

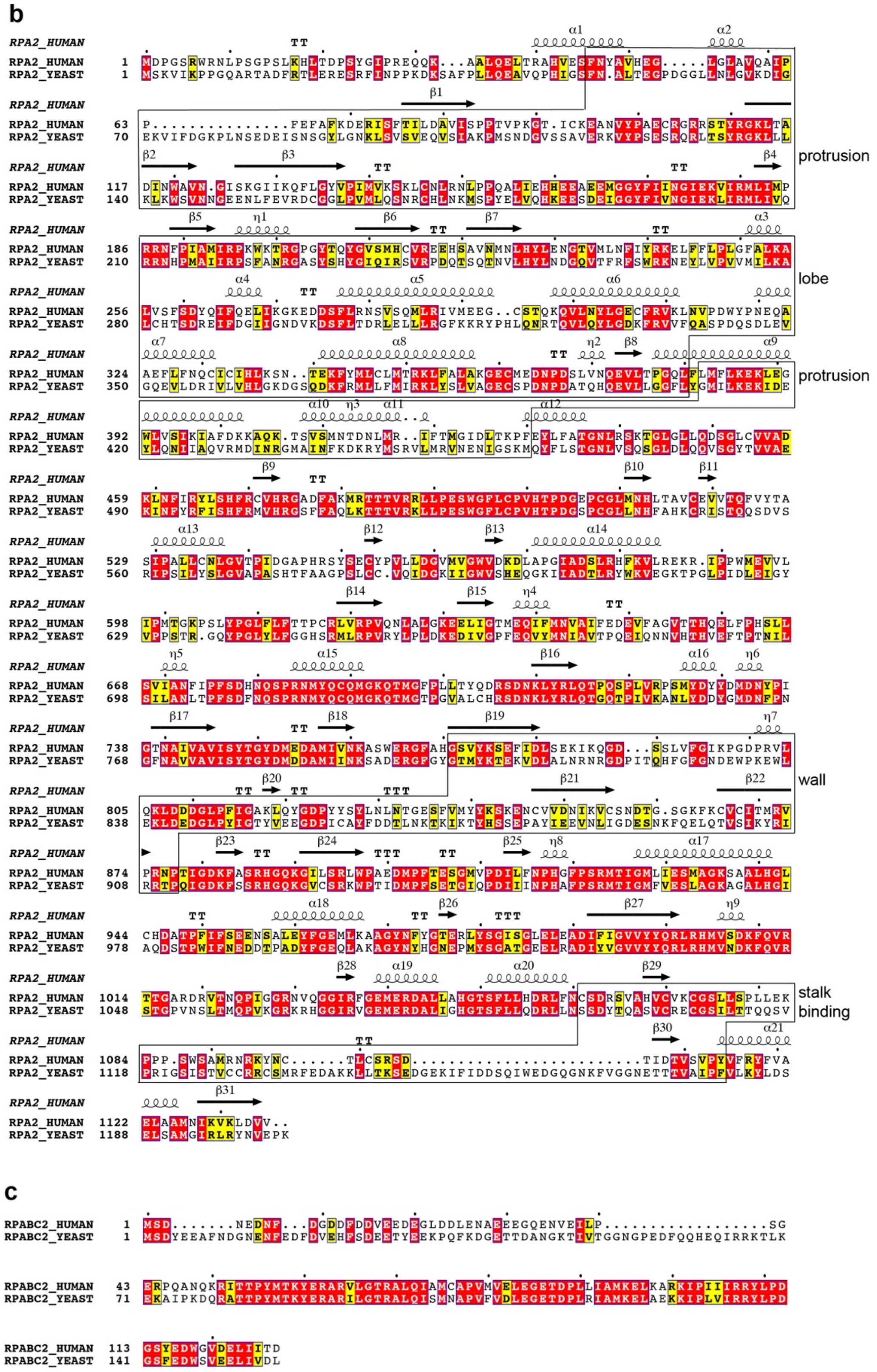

Supplementary Fig. S6 Sequence alignments of human and yeast Pol I subunits.

(a-c) Sequence alignments of RPA1 (a), RPA2 (b), RPABC2 (c) of hPol I and yPol I homologous

subunits, which are also known as A190, A135 and Rpb6 in yeast, respectively. Structural elements/regions of RPA1 and RPA2 are indicated with dark boxes.

Supplementary Table S1 Statistics of cryo-EM data collection, refinement and validation statistics.

	**EC^{pre}**	**EC^{post}**	**EC^{bt}**	**EC^{post}-crosslinking**
EMDB/PDB	31876/7VBA	31877/7VBB	31878/7VBC	-
**Data collection and processing**				
Magnification	130,000	130,000	130,000	36,000
Voltage (kV)	300	300	300	200
Total electron exposure (e^-/Å^2)	~50	~50	~50	~50
Exposure rate (e^-/pix/s)	~8	~8	~8	~21
Number of frames per movie	32	32	32	40
Automation software	SerialEM	SerialEM	SerialEM	SerialEM
Defocus range (μm)	-1.5 to -2.5	-1.5 to -2.5	-1.5 to -2.5	-1.5 to -2.5
Pixel size (Å)	1.054	1.054	1.054	1.1
Symmetry imposed	C1	C1	C1	C1
Micrographs (no.)	3,283	2,074	2,854	505
Total of extracted particles (no.)	1,141,229	618,806	676,465	353,482
Total of refined particles (no.)	382,890	282,280	152,653	127,587
Local resolution range (Å)	6.0-2.0	6.0-2.0	6.0-2.0	6.0-2.0
Resolution Masked 0.143 FSC (Å)	2.89	2.81	3.01	3.89
**Refinement**				
Map sharpening B-factor (Å^2)	100.9	83.6	79.4	-170
Initial model used (PDB code)	5M3F	5M3F	5M3F	
Refinement package	Phenix (real space)	Phenix (real space)	Phenix (real space)	
**r.m.s. deviations**				
Bond lengths (Å)	0.011	0.013	0.013	
Bond angles (°)	1.181	1.035	1.04	
**Validation**				
MolProbity score	2.46	2.5	2.51	
All-atom clashscore	17.66	20.11	19.89	
Rotamers outliers (%)	0.62	1.05	0.19	
Cβoutliers (%)	0	0	0	
CaBLAM outliers (%)	10.1	10.47	10.08	
**B-factors (min/max/mean)**				
Protein	13.95/133.07/53.2	17.91/199.99/72.39	14.30/186.28/79.98	
Ligand	28.47/149.49/84.81	28.78/242.64/147.72	60.15/186.26/136.00	
**Overall correlation coefficients**				
CC (mask)	0.79	0.75	0.74	
CC (peaks)	0.63	0.57	0.56	
CC (volume)	0.76	0.73	0.72	
**Ramachandran plot statistics**				

Favored (%)	81.5	82.35	81.4
Allowed (%)	17.23	16.61	17.72
Disallowed (%)	1.27	1.05	0.89

Supplementary Table S2 RNA and DNA oligonucleotides

**EC$^{post/pre}$ assembly**	
nontemplate DNA	5'-CATTTTGGGCCGCCGGGTTAGGTACTCAGTACTGTCCTCTGGCGAC-3'
template DNA	3'-GTAAAACCCGGCGGCCCAATAACGACTGAGCGACAGGAGACCGCTG-5'
RNA	5'-UGCUGACU-3'
**EC^{bt} assembly**	
nontemplate DNA	5'-CATTTTGGGCCGCCGGGTTAGGTACTCAGTACTGTCCTCTGGCGAC-3'
template DNA	3'-GTAAAACCCGGCGGCCCAATAACGACTGTGCGACAGGAGACCGCTG-5'
RNA	5'-UGCUGACU-3'
**Extension assay**	
nontemplate DNA	5'-GTACTGTCCTCTGGAC-3'
template DNA	3'-ATAACGACTGAGCGACAGGAGACCTG-5'
RNA	5'-FAM-GUGCUGACU-3'
**Cleavage assay**	
nontemplate DNA	5'-CATTTTGGGCCGCCGGGTTAGGTACTCAGTACTGTCCTCTGGCGAC-3'
template DNA	3'-GTAAAACCCGGCGGCCCAATAACGACTGTGCGACAGGAGACCGCTG-5'
RNA	5'-FAM-GUGCUGACU-3'

Supplementary Table S3 Disordered regions that were not modeled in the structure.

RPA1	1-5, 282-289, 315-317, 525-532, 1227-1238, 1302-1312, 1363-1495
RPA2	1-4, 1085-1092
RPAC1	1-7, 344-346
RPAC2	1-20, 129-133
RPA43	1-45, 205-338
PAF53	1-5, 116-419
PAF49	1-7, 158-510
RPA12	1-5, 67-79
RPABC1	1-5, 50-55, 211-215
RPABC2	1-50
RPABC3	1-2, 149-150
RPABC4	1-13
RPABC5	65-67

Supplementary Movie S1 Cryo-EM map and structural model of the Pol I EC^{post}. Color scheme is same as Fig. 1.

Supplementary Movie S2 Structure comparison of hPol I EC and yPol I EC as in Fig. 2.

Supplementary References

1 Neyer, S. *et al.* Structure of RNA polymerase I transcribing ribosomal DNA genes. *Nature* **540**, 607-610, doi:10.1038/nature20561 (2016).

2 Tafur, L. *et al.* The cryo-EM structure of a 12-subunit variant of RNA polymerase I reveals dissociation of the A49-A34.5 heterodimer and rearrangement of subunit A12.2. *Elife* **8**, doi:10.7554/eLife.43204 (2019).

3 Bernecky, C., Herzog, F., Baumeister, W., Plitzko, J. M. & Cramer, P. Structure of transcribing mammalian RNA polymerase II. *Nature* **529**, 551-554, doi:10.1038/nature16482 (2016).

4 Wang, Q. *et al.* Structural insights into transcriptional regulation of human RNA polymerase III. *Nature Structural & Molecular Biology* **28**, 220-227, doi:10.1038/s41594-021-00557-x (2021).

5 Cheung, A. C. & Cramer, P. Structural basis of RNA polymerase II backtracking, arrest and reactivation. *Nature* **471**, 249-253, doi:10.1038/nature09785 (2011).
